# Frequency of *CDH1* Germline Mutations in Non-Gastric Cancers

**DOI:** 10.3390/cancers13102321

**Published:** 2021-05-12

**Authors:** Giulia Massari, Francesca Magnoni, Giorgio Favia, Nickolas Peradze, Paolo Veronesi, Carlo La Vecchia, Giovanni Corso

**Affiliations:** 1 Division of Breast Surgery, European Institute of Oncology (IEO), Istituto di Ricovero e Cura a Carattere Scientifico (IRCCS), 20141 Milan, Italy; giulia.massari@ieo.it (G.M.); francesca.magnoni@ieo.it (F.M.); giorgio.favia@ieo.it (G.F.); nickolas.peradze@ieo.it (N.P.); paolo.veronesi@ieo.it (P.V.); 2 Department of Oncology and Hemato-Oncology, University of Milan, 20122 Milan, Italy; 3 Department of Clinical Sciences and Community Health, University of Milan, 20133 Milan, Italy; carlo.lavecchia@unimi.it

**Keywords:** *CDH1* mutation, diffuse gastric cancer, Lobular breast cancer, hereditary cancer

## Abstract

**Simple Summary:**

Diffuse gastric cancer is the hallmark of the hereditary diffuse gastric cancer syndrome related with the E-cadherin germline mutations. Other cancers (non-gastric) are described in association with the *CDH1* gene germline alterations. In this study, we aimed to assess the overall frequency of *CDH1* mutations in non-gastric tumors reported in literature so far.

**Abstract:**

Hereditary Diffuse Gastric Cancer (HDGC) is a complex inherited syndrome caused by *CDH1* germline mutations. DGC is the hallmark cancer of this genetic predisposition, but several other cancers are associated with these *CDH1* mutations. In this review, we revised all studies reporting *CDH1* mutations in non-GC patients. The selected studies included: (a) families aggregating with GC and other cancers, both, and (b) families presenting only non-gastric tumors association. Among non-gastric tumors, our results show that *CDH1* mutations are most frequently identified in breast cancer. The frequency of missense mutations is higher in the non-GC group, as the age at diagnosis in this group. Moreover, the predominant *CDH1* mutation affects the extracellular domain. Our data suggest that *CDH1* genetic testing should be considered also in other cancers, especially breast tumors.

## 1. Introduction

Hereditary Diffuse Gastric Cancer (HDGC) is a rare autosomal dominant syndrome that is associated with an increased risk of two major cancer types: diffuse gastric cancer (GC) and lobular breast cancer (LBC) [1]. In particular germline alterations of the E-cadherin gene, *CDH1*, occur in about 40% of all HDGC cases screened [2], with an estimated lifetime risk of diffuse gastric cancer (DGC) of 70% in men and 56% in women, and a cumulative incidence of LBC in women of around 42% [3]. The need for correct management for this inherited cancer predisposition created the International Gastric Cancer Linkage Consortium (IGCLC) in 1999 [4]. Since then, international multidisciplinary groups have been establishing and updating the clinical criteria for early disease diagnosis and for the detection of patients who should be eligible for germline *CDH1* genetic screening. The more recent revised guidelines establish HDGC as any family with one of the following clinical criteria: (1) families with two or more certain cases of GC at any age, one with documented DGC; (2) personal history of DGC before the age of 40 years; (3) personal or family history of DGC and LBC, with one diagnosis before 50 years of age [2]. Although DGC is the index tumor type in HDGC, other malignant cancers [5], and congenital malformations [6], have been reported in families affected by this syndrome. For that reason, LBC, oral facial clefts, colorectal carcinomas (CRC), and other cancers, have been suggested as suitable for *CDH1* screening and early detection of HDGC [2]. However, except for LBC, their inclusion in HDGC clinical definition is not yet supported due lack of robust data. 

With this in mind, we have reviewed the literature for all *CDH1* germline mutations in the non-DGC spectrum, as well LBC, and other epithelial cancers.

## 2. Methods

We reviewed all *CDH1* germline mutations reported in MEDLINE (https://www.nlm.nih.gov/medline/medline_overview.html, accessed on 12 March 2021) in individuals with diagnosed GC or other cancers, from 1998 to 2020, including original reports and literature reviews in English. The following terms were used for the literature search: E-cadherin; *CDH1* gene; germline mutation; genetic screening; HDGC; IGCLC; familial GC; diffuse histotype; lobular histotype; BC; Maori kindred and prophylactic gastrectomy. This analysis was limited to studies involving subjects with HDGC, early-onset GC, and unselected GC patients screened for *CDH1* germline mutations, LBC, CRC, prostate cancer (PC), ovarian cancer (OC), abdominal carcinosis (Ca), thyroid cancer (ThC), tongue cancer (ToC), in which at least one likely pathogenic, VUS or pathogenic *CDH1* variant was identified. Mutation types were classified as missense, splice site, deletion, insertion, and nonsense alterations.

We considered two groups: (a) families aggregating with GC and non-GC both, and (b) families associating with other tumors.

## 3. Results 

Table 1 resumes *CDH1* germline mutations identified in the non-GC population. Other details on the frequency of *CDH1* mutations in HDGC syndrome were previously described in our recent study [7]. Twenty-three families presented non-GC in their history, but only other tumor types. Instead, fifteen families aggregated with GC and other tumors, both. The mean age at diagnosis for individual *CDH1* mutant carriers with GC was 40.6 years (range 21–79), and for other cancers 50.6 years (range 23–63).

### 3.1. Other Types of Cancer

**Figure 1:**
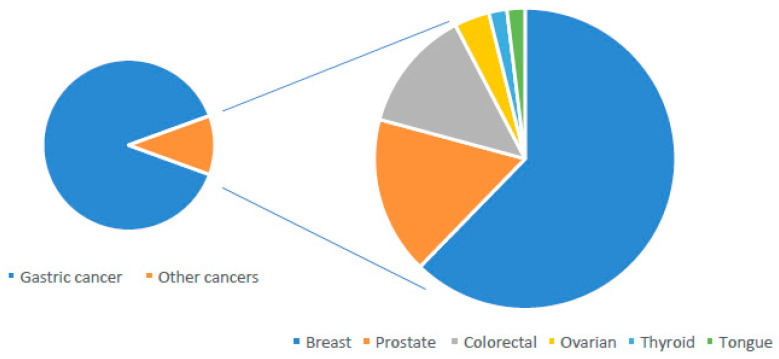
Frequency of *CDH1* germline mutations in gastric cancer and other cancers (cancers in detail are shown in the second pie).

We identified 54 *CDH1* germline mutations in non-gastric tumors. The most frequent tumor associated with *CDH1* germline mutations was BC (33/54, 61.1%), following prostate cancer (PC) (9/54, 16.7%), CRC (7/54, 12.9%), abdominal carcinosis (Ca) (2/54, 3.7%), ovarian cancer (OC) (1/54, 1.8%), thyroid cancer (ThC) (1/54, 1.8%), and tongue cancer (ToC) (1/54, 1.8%) (Figure 1). Regarding BC, interestingly we observed that in non-GC families, BC with *CDH1* mutations occurs with a frequency of 42.6% (23/54), and in mixed families 18.5% (10/54). Thus, let us suppose that BC with *CDH1* mutation aggregates more frequently with an independent pathway. 

### 3.2. Type of Mutations

**Figure 2:**
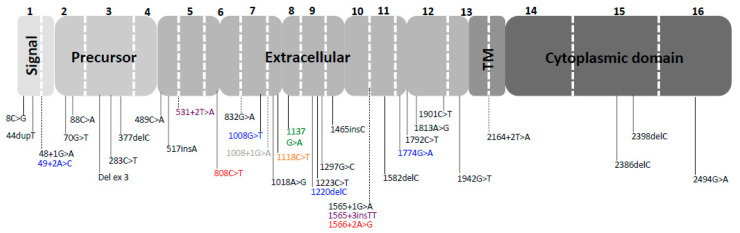
Mutation distribution in the different sites of *CDH1* gene (SG: signal, TM: transmembrane, red: prostate cancer, black: breast cancer, orange: ovarian cancer, blue: colorectal cancer, purple: abdominal carcinosis, green: tongue cancer, and grey: thyroid cancer).

Considering the 54 *CDH1* germline mutations identified in non-GC, we verified that the frequency type of mutations were as follows: missense 48.2% (26/54), splice site 18.5% (10/54), deletion 11.1% (6/54), insertion 11.1% (6/54), and nonsense 11.1% (6/54), respectively. With regard to the localization of the mutations identified in non-GC cancers, we verified that the majority of *CDH1* mutations affected the extracellular domain (31/54, 57.4%), followed by precursor (13/54, 24.1%), signal (6/54, 11.1%), cytoplasmic (3/54, 5.6%), and transmembrane (1/54, 1.8%) domains (Figure 2).

Considering the ClinVar classification, and the submitted mutations, we identified only the S270A mutation classified as “VUS”. Most of the collected mutations were not submitted at the ClinVar platform.

## 4. Discussion

After lung and colorectal cancer, GC remains the third cause of cancer deaths worldwide, with about 1.2 million cases and almost one million deaths worldwide, and still is the leading cause of cancer and cancer death in selected low-income areas [27]. The substantial variations over geographic areas and declines in incidence and mortality over the last few decades indicate that most GCs have an environmental origin, Helicobacter pylori infection being by far its main cause [28]. However, there is a well identified and quantified family clustering of GC, with relative risks around two for a family history of GC in first degree relatives, after accounting for recognized environmental factors, with generally stronger associations at a younger age [29,30]. Therefore, understanding and quantifying the key components of familial and genetic factors on the different pathologic types of GC remains of key interest for understanding pathogenesis and defining early diagnosis and, hence, management. Furthermore, it might contribute to increase quality of life and the patient’s involvement in clinical decision-making [31,32,33].

The *CDH1* gene (OMIM No. 192090) is located on chromosome 16q22.1 and encodes for the E-cadherin protein [34]. This macro-molecule is a trans- membrane glycoprotein expressed on epithelial tissue and is responsible for calcium-dependent, cell-to-cell adhesion [24]. E-cadherin protein is critical for establishing and maintaining polarized and differentiated epithelia through intercellular adhesion complexes. The human E-cadherin function is to suppress cell invasion; in fact, its deregulation is correlated with the infiltrative and metastatic ability of the tumor [23], with the consequent loss of cell adhesion and concomitant increase in cell motility [35]. In human samples, somatic *CDH1* alterations are associated with poor survival and worse prognosis in gastric cancer patients [29].

In 1998, Guilford et al. identified a large family from New Zealand with multiple cases of DGC that were carriers of a causative germline mutation in the E-cadherin gene [36]. Thereafter, several publications emerged, confirming the autosomal dominant pattern of inheritance associated with germline mutations of the *CDH1* gene. To date, more than 500 germline mutations have been identified in DGC families. The most common types of mutations are missense (23%), nonsense (22%), deletions (22%) or insertions (10%), and splice site (21%) [7].

In this study, firstly, we reviewed the frequency of *CDH1* germline mutations in non-gastric tumors, and we identified 54 alterations. The major findings of this study were the following: (a) *CDH1* germline mutations excluding GC are found predominantly BC (60% of all non-GC cancers); (b) the mean age at diagnosis was higher in the non-GC cancer (50.6 year), compared to GC (40.6 year); (c) the majority of mutations were localized in the cytoplasmic domain (Figure 2). 

### 4.1. First Point

Recent studies demonstrated that LBC might be the first manifestation of the HDGC syndrome, even in the absence of DGC cases in the family. It was supposed that families with LBCs carrying *CDH1* germline mutations cluster as independent inherited syndrome. The concept of hereditary lobular breast cancer (HLBC) is very recent [5], because some authors identified pathogenic *CDH1* mutations in women with LBC [37,38], and without GC family history. To date, we do not know if asymptomatic individuals in these families will develop gastric cancer later, the cumulative risk of developing GC in these women or their relatives with *CDH1* mutations is unknown; it is possible that in this contest the penetrance risk for GC is lower or absent. 

In cases of the classic HDGC syndrome, individuals carrying pathogenic mutation in *CDH1* have about a 70% risk of developing DGC and women have an additional LBC risk of approximately 40%, by the age of 80 years [3]. Although some authors stated that the *CDH1* variant carriers lifetime risk of developing invasive BC is similar to that of *BRCA* mutation carriers [39], the real risk of developing LBC in absence of a clear HDGC predisposition remains undetermined. 

### 4.2. Second Point

BC, as well as other non-GCs associated with *CDH1* mutations, could be a non-early manifestation of the complex HDGC syndrome. We observed, in fact, that the main age at diagnosis of BC, as well of other non-GCs, is higher. The pathway of *CDH1* deregulation during breast and gastric carcinogenesis is different and this could explain also the different clinical manifestations. In BC, the presence of a *CDH1* mutation can alter the E-cadherin function, causing a decreased cell–cell adhesion and an increased cell proliferation, so-called lobular hyperplasia. Subsequently, a second-hit *CDH1* inactivation causes the loss of E-cadherin expression and, after, alters organization of the lobule. During this event, deregulated cells emerge and accumulate in the lobules creating a lobular intraepithelial neoplasia pattern. Finally, cancer cells disrupt the basement membrane and invade surrounding breast tissue, a tumor stage that is classified as invasive lobular carcinoma [5]. In *CDH1* gastric carcinogenesis, the early-stage of HDGC is characterized by multiple foci of invasive (T1a) signet-ring cell (diffuse) carcinoma in the superficial gastric mucosa, with no nodal metastases. In situ signet-ring cell carcinoma and pagetoid spread of signet ring cells are recognized precursors (Tis) to T1a signet ring cell carcinoma [40]. Gastric carcinogenesis associated with *CDH1* germline mutations seems more “aggressive” than LB tumorigenesis, in which missense mutations predominate. It is possible that the penetrance of cancer risk from pathogenic missense *CDH1* variants is lower than that from truncating mutations, and maybe other factors (hormonal?) play a progressive synergic role with *CDH1* missense mutations in BC development (as in other cancers).

### 4.3. Third Point

The accumulation of mutations in the cytoplasmic domain is an interesting point. The cytoplasmic domain of E-cadherin has a crucial role in its function, because it supports the assembly of a complex of cytosolic proteins, including catenins, which provide anchorage to the actin cytoskeleton to form stable cell–cell contact [41]. Thus, the cytoplasmic domain represents a vulnerable point due to its intrinsic nature and the presence of mutations affecting this point confers a dangerous alteration of the E-cadherin protein function.

### 4.4. Limitations of the Study

(a) We have to consider that *CDH1* gene in BC, as well in CRC, play a minor role; *BRCA1/2* and mismatch repair proteins (*MSH2, MSH6, MLH1, PMS2*) exert a major impact in their carcinogenesis. The possibility to find a *CDH1* mutation, in accord with our data, is very low in the non-GC group. At this moment, it is not clear if the identification of *CDH1* mutation in the non-GC group is “casual” or associated with a possible minor pathway in non-GC tumorigenesis. We suppose that in BC the identification of *CDH1* mutation is not a casual or an “incidental finding”, some data demonstrated that there is mutual exclusion of *CDH1* and *BRCA* germline mutations in the pathway of hereditary BC [42].

(b) Another limitation of this study is the missing information about ClinVar classification. Unfortunately, not all mutations were submitted at the ClinVar platform, and this work should be completed to better clarify the potential pathogenic role of *CDH1* mutations reported in the current study.

(c) The motivation to perform *CDH1* genetic screening in such populations, without apparent indications, is unknown. Unfortunately, the authors did not clarify this point. This missing information represents another limitation of this study.

## 5. Conclusions

In conclusion, in comparison with our previous study [7], our results suggest that about 7% of the overall *CDH1* mutations are present in non-gastric tumors. The majority of mutations are identified in BC, and the age at diagnosis is higher in other cancers, in comparison to GC group. It is plausible that non-GC cancers are a late manifestation of the HDGC syndrome. Mutations affect predominantly the cytoplasmatic domain of *CDH1* gene, a vulnerable place that exerts a pivotal role in the cell–cell adhesion and polarization. *CDH1* missense mutations are more frequent in non-gastric tumors (48.2%), and other factors could play a synergistic role with missense mutations in the development of non-GCs. At this moment, the value of *CDH1* testing in non-GC cancers other than BC may not be high, given the paucity of evidence. A prospective study may be of more value in changing clinical practice.

## Figures and Tables

**Table 1:** List of *CDH1* germline mutations identified in non-gastric cancers. In BC series, histotype was not reported (abbreviations: HGVS: Human Genome Variation Society nomenclature; DGC: diffuse gastric cancer; OC: other cancer; UK: United Kingdom).

First Author	Country	Type	HGVS	Localization	Protein	*CDH1*_DGC	*CDH1*_OC	OCs
Ikonen 2001 [8]	Finland	Missense	808T>G	Ex_6	S270A	-	8	PC
Oliveira 2002 [9]	Europe	Insertion	44dupT	Ex_1	-	1	3	CRC(2), LBC(1)
Lynch 2008 [10]	USA	Non sense	70G>T	Ex_2	-	5	3	BC
Xie 2011 29]	France	Non sense	283C>T	Ex_3	-	-	3	LBC
Chen 2013 [11]	China	Missense	1018A>G	Ex_8	T340A	-	3	LBC
Salahshor 2001 [12]	Sweden	Missense	1774G>A	Ex_12	A592T	-	2	CRC
Katona 2020 [13]	USA	Splice site	1566+2A>G	In_10	-	1	1	PC
Gullo 2018 [14]	Portugal	Missense	1901C>T	Ex_12	A634V	7	1	LBC
López 2016 [15]	Spain	Deletion	1220delC	Ex_9	-	2	1	CRC
Bardram 2014 [16]	Denmark	Insertion	1565+3insTT	In_10	-	5	1	Ca
More 2007 [17]	Caucasian	Splice site	49+2A>C	In_1	-	3	1	CRC
	Caucasian	Splice site	1137G>A	Ex_8	-	2	1	ToC
Kluijt 2012 [18]	Unknown	Non sense	489C>A	Ex_4	-	2	1	LBC
Guilford 2010 [19]	Europe	Non sense	70G>T	Ex_2	-	3	1	BC
Keller 2004 [20]	Germany	Deletion	377delC	Ex_3	-	2	1	LBC
Frebourg 2006 [6]	Caucasian	Splice site	531+2T>A	In_4	-	3	1	Ca
Guilford 2010 [20]	Maori	Splice site	1008G>T	Ex_7	-	9	1	CRC
Oliveira 2002 [9]	Pakistan	Splice site	832G>A	Ex_6	-	3	1	LBC
Lynch 2008 [10]	USA	Non sense	1792C>T	Ex_12	-	1	1	BC
Masciari 2007 [21]	USA	Insertion	517insA	Ex_4	-	-	1	LBC
Schrader 2011 [22]	USA	Splice site	1565+1G>A	In_10	-	-	1	BC
	Canada	Missense	8C>G	Ex_1	P3R	-	1	BC
	Canada	Missense	88C>A	Ex_2	P30T	-	1	BC
	Canada	Missense	88C>A	Ex_2	P30T	-	1	BC
	Canada	Missense	1223C>T	Ex_9	A408V	-	1	BC
	Canada	Missense	1297G>C	Ex_9	D433N	-	1	BC
	Canada	Missense	1813A>G	Ex_12	R605G	-	1	BC
	Canada	Missense	2494G>A	Ex_16	V832M	-	1	BC
Petridis 2014 [23]	UK	Splice site	48+1G>A	Ex_1	-	-	1	LBC
	UK	Insertion	1465insC	Ex_10	-	-	1	LBC
	UK	Missense	1942G>T	Ex_13	E648X	-	1	LBC
	UK	Deletion	2398delC	Ex_15	-	-	1	LBC
Benusiglio 2013 [24]	France	Splice site	2164+2T>A	In_13	-	-	1	LBC
	France	Deletion	del_ex_3	Ex_3	-	-	1	LBC
	France	Splice site	1008+1G>A	In_7	-	-	1	ThC
	France	Deletion	2386delC	Ex_15	-	-	1	LBC
Xie 2011 [25]	France	Deletion	1582delC	Ex_11	-	-	1	LBC
Manchana 2019 [26]	Thailandia	Missense	1118C>T	Ex_8	P373L	-	1	OC
